# Electrochemical Strips Modified with Zeolites Embedding Silver Clusters for Versatile (Bio)Systems

**DOI:** 10.1021/acs.analchem.4c03654

**Published:** 2024-10-29

**Authors:** Cecilia García-Guzmán, Ada Raucci, Eduardo Coutino-Gonzalez, Eden Morales-Narváez, Stefano Cinti

**Affiliations:** † Department of Pharmacy, University of Naples “Federico II”, 80131 Naples, Italy; ‡ Centro de Investigaciones en Óptica, A. C., Loma del Bosque 115, Lomas del Campestre, León, Guanajuato 37150, Mexico; § Materials & Chemistry Unit (MatCh), VITO, Flemish Institute for Technological Research, Boeretang 200, Mol B-2400, Belgium; ∥ Biophotonic Nanosensors Laboratory, Centro de Física Aplicada y Tecnología Avanzada (CFATA), Universidad Nacional Autónoma de México (UNAM), Querétaro 76230, Mexico; ⊥ Bioelectronics Task Force, University of Naples Federico II, Via Cinthia 21, Naples 80126, Italy; # Sbarro Institute for Cancer Research and Molecular Medicine, Center for Biotechnology, College of Science and Technology, Temple University, Philadelphia, Pennsylvania 19122, United States

## Abstract

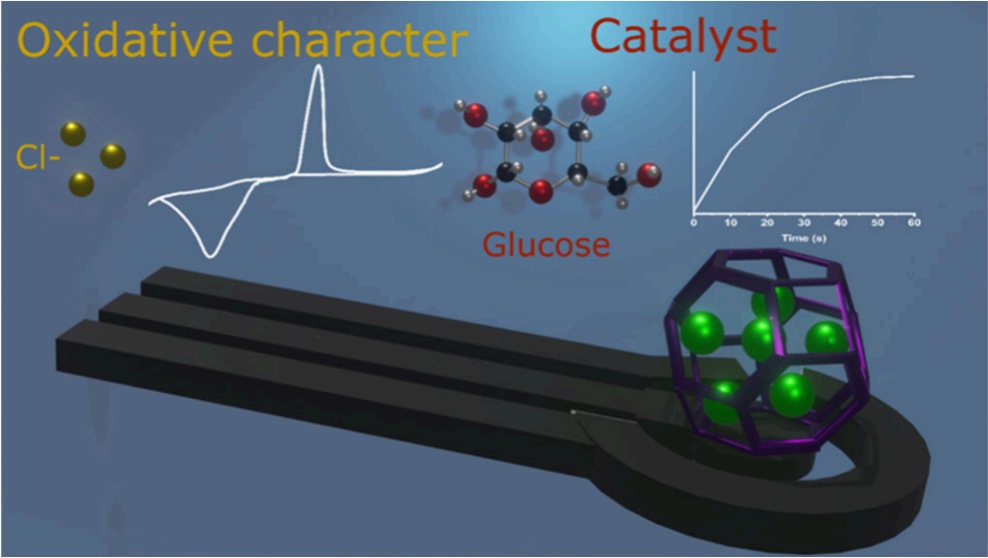

Disposable biosensors provide high portability and advantageous response time, which are crucial in the next generation of analytical platforms. Herein, we report on zeolites embedding silver clusters as an electroactive material that enhances the electrode–analyte interaction in disposable electrochemical strips made of plastic or paper. The resulting analytical platform effectively immobilizes analytes and facilitates electron exchange transfer. Two detection mechanisms were explored in the electrodes decorated with zeolites embedding silver clusters. The oxidative–reductive character of zeolites embedding silver clusters was applied for chloride ion detection, which is suitable for environmental analysis. Additionally, the electrocatalytic activity of the composite was applied in glucose sensing in the presence of glucose oxidase, demonstrating the versatility of the composites for detecting molecules of a different nature (clinically or environmentally relevant) without further modification. Our approach contributes to the development of simple and low-cost modification procedures for integrating electroactive materials into miniaturized electrochemical sensors for portable platforms and point-of-care devices.

## 1 Introduction

The field of sensors and biosensors has significantly advanced through the integration of traditional transduction architectures (i.e., electrochemical, optical, or mechanical) with innovative materials.^1,2^ These materials enhance analytical performance for specific targets by improving sensitivity, specificity, portability, easiness, sustainability, affordability, quickness of response acquisition, low cost, and speed of detection.^2,3^ For instance, nanomaterials often exhibit higher conductivity and reactivity compared to their bulk counterparts due to their high surface-to-volume ratio and increased active sites compared with their bulk counterparts.^4,5^

Zeolites are porous materials that are characterized by a tetrahedral crystalline structure of aluminosilicate, exhibiting channels and cavities. The replacement of Si^4+^ by Al^3+^ atoms in the framework structure generates an intrinsic negative charge that is compensated by the introduction of counterbalancing cations such as Na^+^, K^+^, and Li^+^ in the extra-framework structure, allowing the incorporation of different guess species (metal ions, dyes, organic molecules) in the zeolite’s cavities by direct interchange with the counter cations in a selective way due to their specific porous size and hydrophobicity, determined by Si/Al ratio. Zeolites boast several advantages including high pore volume/surface area, effective biomolecule absorption, chemical and thermal stability, and biocompatibility.^6,7^ Moreover, the exploitation of their porosities enhances interaction with target species, setting the foundation for advanced electrochemical systems.^6^ Additionally, zeolites serve as an ideal matrix for ion exchange due to their uniform pore size, abundant active sites, and resistance to extreme conditions. They have been successfully utilized in enzymatic assays as immobilization matrices for various enzymes, such as glucose oxidase, urease, acetylcholinesterase, glutamate oxidase, acetylcholinesterase, and horseradish peroxidase (HRP), among others.^8−10^ This approach has been shown to improve analytical performance by 20–30% through strong immobilization capacity and high electrostatic attraction, while simplifying the process by avoiding toxic reagents such as glutaraldehyde, typically used in covalent-cross-linking procedures.^8,11,12^

The ion exchange property of zeolites can be leveraged to synthesize active metal clusters. For instance, silver clusters have been synthesized inside zeolite cavities, resulting in composites with high stability and catalytic activity.^13^ Despite the potential benefits of silver clusters within zeolites in electroanalytical systems, reports on their application remain limited. Among these, silver clusters embedded in zeolites have been combined with carbon paste to manufacture working electrodes for tryptophan amino acid in milk, enhancing electron transfer by reducing the oxidation reaction’s overpotential.^14^ Additionally, silver clusters embedded within zeolites have been used to modify glassy carbon electrodes for the detection of sulfadiazine antibiotic in seawater and α-fetoprotein in human serum,^15,16^ improving electrical conductivity and electroactive surface area, toward accurate, synergetic, selective, and stable electrochemical sensors. Given these advantages, zeolite-embedded silver clusters might play a crucial role in electrochemical analysis and biosensing, by enhancing conductivity, electrocatalytic activity, and surface-to-volume ratio.^16^

Due to their exceptional properties, mainly demonstrated in optical systems for biosensing,^17^ we hypothesize that silver cluster–zeolite composites could play a significant role when combined with portable and miniaturized electrochemical strips, specifically screen-printed electrodes, to create a novel type of (bio)sensor. In this work, silver cluster–zeolite composites have been applied in combination with different types of electrochemical strips, including polyester- and paper-based substrates. As a proof of concept, the effectiveness of the electrodes modified with silver cluster–zeolite composites has been characterized in two scenarios: first, its oxidative–reductive potential for chloride ions detection, analyzing the established electrochemistry of silver in the presence of chloride ions, i.e., AgCl + e^–^ → Ag + Cl^–^,^18,14^ demonstrating satisfactory analytical performance and adaptability with both traditional plastic-based and novel paper-based substrates. Furthermore, the electrocatalytic capacity of the designed silver cluster–zeolite composite has been satisfactorily evaluated in combination with glucose oxidase enzyme for glucose detection, demonstrating an enhancement in the current signal due to the composite, which improved the analytical performance of the biosensor. High selectivity for glucose detection was also observed when evaluating other clinically relevant biomolecules present in human biofluids, confirming the silver cluster–zeolite composite as a promising material for novel biosensing applications. Additionally, the integration of silver cluster–zeolite composite with paper-based substrates offers a greener/eco-friendly platform for their use in decentralized areas, providing a robust, cost-effective, simple to operate and accessible solution while reducing the environmental impact associated with production and waste management.^19^

This Technical Note is focused on characterizing and evaluating the combination of zeolites embedding silver clusters in electroanalytical systems. For the first time, this composite has been demonstrated as an active material that enhances the analytical performance of printed strips, highlighting its versatile role as an electron exchange transfer facilitator and electrocatalyst. This might represent the starting point to extend the adoption of silver cluster–zeolite composite toward integration with other (bio)systems for the detection of clinically and environmentally relevant molecules, such as inorganic species and biomolecules.

## 2 Experimental Section

Silver nitrate (AgNO_3_) (99.9999%), potassium chloride (KCl) (>99%), hydrogen peroxide (solution 30 wt % in water), glucose oxidase from *Aspergillus niger* G7141, glucose, and phosphate buffer saline (PBS) tablets (140 mM NaCl, 10 mM phosphate buffer, 3 mM KCl pH 7.4) were purchased from Merck. Faujasite X (FAUX) Si/Al ratio 1.3 was obtained from Clariant. A 100 mL alumina ball mill jar EQ-AJ-80 and 2 mm diameter alumina milling balls were purchased from MTI CORP Interlab. Ag/AgCl and carbon inks were purchased from SunChemical. Polyester Autostat HT5 was kindly provided by McDermid, and the paper-based chromatographic substrate (Whatman No. 1) was purchased from Merck.

### 2.1 Screen-Printed Electrodes

According to previously described methods,^20^ flexible electrodes were produced onto two substrates: polyester and chromatographic Whatman No. 1 filter paper. To create hydrophobic barriers in paper-based strips, the working area was printed using a wax printer and thermally treated in an oven for 1 min at 80 °C to allow the penetration of the wax into the paper pores. Subsequently, for both the polyester- and paper-based systems, the three-electrode system was screen printed using Ag/AgCl ink for the connections and the reference electrode. It was thermally treated for 30 min (the polyester platform at 100 °C and the paper-based platform at 80 °C). Subsequently, carbon ink was printed as the working and counter electrodes and thermally treated as previously done for silver ink. Finally, an adhesive tape was used to delimit the working area of polyester electrodes, while the working area of the paper-based electrodes was delimited by the printed wax.

### 2.2 Silver Clusters into Zeolite Synthesis

Silver clusters confined in zeolites were synthesized according to previously reported methods.^17^ Briefly, the zeolites were reduced in size to prepare a colloidal suspension. To this end, 3 g of faujasite X (FAUX) was put in a high vibrational miller in 6 intervals of 10 min of milling and 5 min of break recovering the sample by centrifugation at 600 rpm for 30 min; it was then washed two times with deionized water and dried in an oven at 110 °C for 3 h. Next, 500 mg of this milled FAUX was suspended in a solution of silver nitrate (6.8876 mM) in deionized water and stirred overnight in darkness. Then, zeolites were collected by centrifugation at 600 rpm for 30 min, washed twice with deionized water, and dried for 2 h at 80 °C. To generate the silver clusters, a thermal reduction was carried out by making a temperature ramp of 5 °C/min from room temperature to 110 °C, and it was maintained for 2 h at 110 °C. After, a temperature ramp of 5 °C/min was implemented from 110 to 450 °C and maintained for 2 h at 450 °C. The obtained product was named FAUX-Ag.

### 2.3 Modification of the Working Electrode

**Scheme 1:**
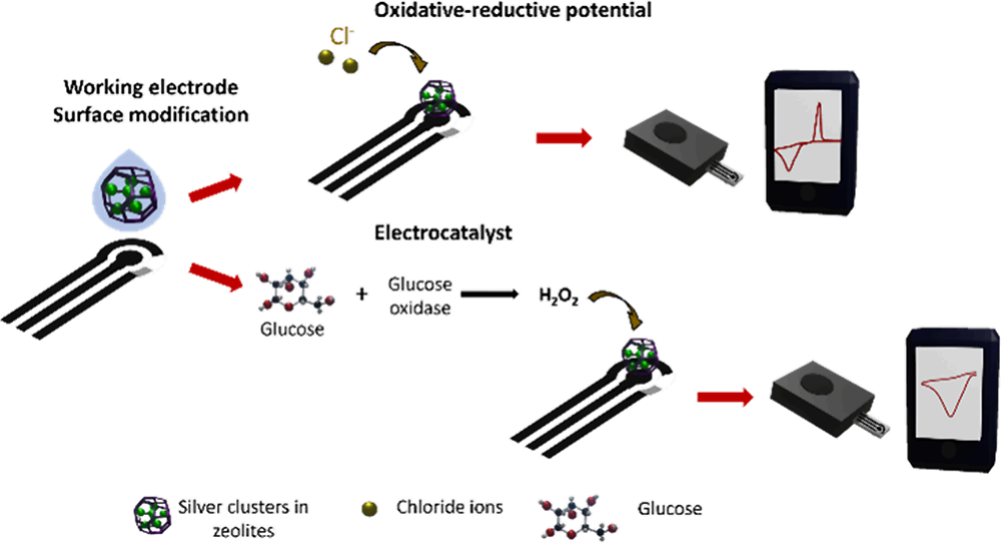
Electrode Modification and Sample Analysis Representation of the procedure to modify the working electrode surface and the sample evaluation using a portable potentiostat by two different mechanisms, the oxidative-reductive potential for chloride ions sensing and electrocatalyst for glucose detection.

The working electrode was readily modified by drop-casting; in particular, 4 μL of FAUX-Ag suspension was dropped at the selected concentration (3, 6, and 9 mg/mL) and dried at room temperature as shown in Scheme 1.

### 2.4 Polyester and Paper-Based Substrate Evaluation

Morphological images were obtained in a Scanning Electron Microscopy JEOL JSM-7800F, and all the electrochemical measurements were carried out by drop casting 80 μL of KCl solution (0–30 mM) in the working area of the electrode connected to a portable Sensit/Smart potentiostat (PalmSens, Netherlands).

### 2.5 H_2_O_2_ Evaluation

After connecting the electrode to the potentiostat, a drop of 80 μL of the desired concentration of H_2_O_2_ solution (0–50 mM) was deposited in the working area, and the respective measurements were carried out in these conditions.

#### 2.5.1 Glucose Evaluation

Glucose was evaluated by producing H_2_O_2_ through an enzymatic reaction with glucose oxidase. To this end, 76 μL of glucose oxidase at the explored concentrations (0.1, 1, and 10 mg/mL) was added in a microtube, and 4 μL of glucose diluted at different concentrations (0–10 mM) was added in the same microtube. After 5 min of the reaction, the total volume in the eppendorf tube was dropped and deposited in the working area of the electrode previously connected to the potentiostat.

## 3 Results and Discussion

### 3.1 Oxidative–Reductive Character of the Composite: Chloride Ions Sensing

**Figure 1:**
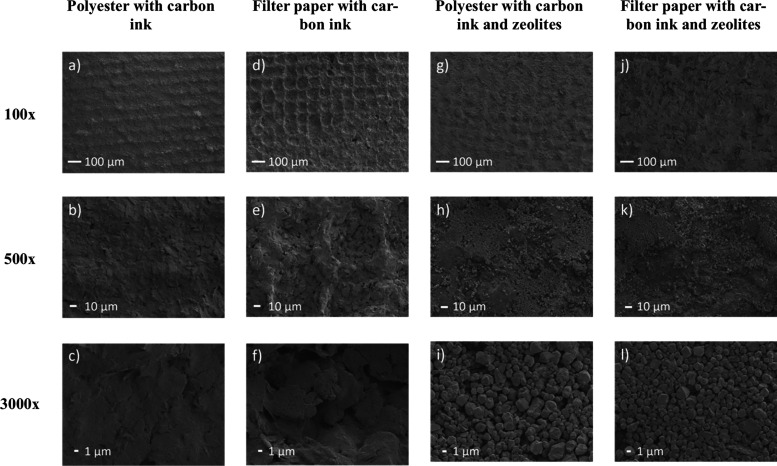
SEM images of carbon working electrodes printed on polyester and filter paper substrates before and after surface modification with silver clusters into zeolites at 3 different magnifications 100×, 500×, and 3000×. Polyester with carbon ink (a–c) before zeolite deposition and (g–i) after zeolite deposition. Filter paper with carbon ink (d–f) before zeolite deposition and (j–l) after zeolite deposition.

This study investigated two commonly used substrates for screen-printing electrodes: polyester and chromatographic paper-based. Polyester is flexible, highly resistant to mechanical stress and does not exhibit absorption of sample,^21^ while the paper-based substrate, composed of cellulose, has intrinsic porosity that allows for species adsorption and acts as a filter in complex matrices.^22,23^ To obtain a complete characterization of the systems, both platforms were morphologically evaluated before and after modification with zeolites containing silver clusters, as reported in Figure 1.

Due to the smooth surface of polyester, the deposition of carbon-based conductive ink was relatively homogeneous with small roughness due to the mesh used for the screen-printing technique (Figure 1a–c). In contrast, the fibrous nature of chromatographic paper led to a nonhomogenous carbon ink deposition, generating a rough electrode (Figure 1d–f). These differences in the carbon ink deposition generate differences in the surface modification with zeolite particles since the homogeneous carbon ink deposition on polyester allows zeolite deposition in a few layers (Figure 1g–i), resulting in an average of 45 ± 2 particles per 10 μm^2^ and a mean particle size of 1.4 ± 0.5 μm (200 particles) on the surface. In comparison, filter paper exhibits a heterogeneous deposit in several layers with the smallest particles deeper within the layers and large particles on the surface (Figure 1j–l), averaging 35 ± 1 particles per 10 μm^2^ with a mean size of 1.8 ± 0.5 μm (200 particles) on the surface.

As observed in Figure S1 of the SI, EDS images confirm that silver clusters are embedded within the zeolite particles distributed across the working electrode surface. Further characterization of the material is detailed in previous articles,^24−26^ highlighting their high stability and their capacity to respond to environmental stimuli.

Subsequently, to evaluate the effect of particle deposition on the working electrode surface on polyester- and paper-based platforms, the electrochemical behavior of modified electrodes was assessed. The zeolite structure containing positively charged silver clusters can selectively interact with ions that are internalized into zeolite pores, such as chloride ions with a diameter of 3.7 Å.^27^ The main electrochemical reaction that enables the detection of chloride ions is an oxidation of silver ions, converting Ag^0^ to Ag^+^ in the presence of chloride ions to form AgCl. A similar approach has also been reported in the literature for the fluorometric detection of other molecules, such as formaldehyde.^28^

**Figure 2:**
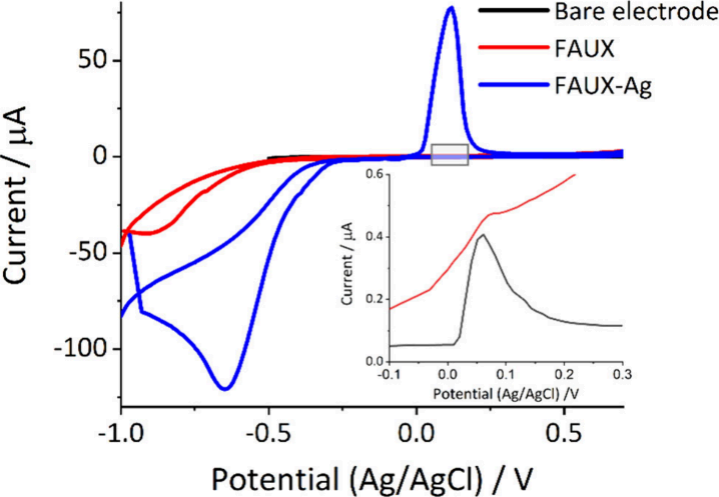
Comparison of the bare working electrode, working electrode modified with FAUX (6 mg/mL), and working electrode modified with FAUX-Ag (6 mg/mL) in the presence of 10 mM KCl. Cyclic voltammetry scan rate, 0.05 V/s. All measurements were performed in triplicate using different electrodes.

Herein, chloride ion detection was conducted to evaluate the efficacy of silver clusters confined within Faujasite X zeolites (FAUX-Ag) as an electroactive booster, resulting from the modification of the working electrode area. As shown in Figure 2, cyclic voltammetry experiments were performed by comparing the oxidative signal in the presence of bare electrodes, electrodes decorated with FAUX (6 mg/mL), and electrodes decorated with FAUX-Ag (6 mg/mL) as the complete composite because naked metallic clusters present aggregation on polyester substrates in the presence of chloride ions. A peak centered at 0.1 V (vs Ag/AgCl), associated with the silver oxidation reaction, was observed only on electrodes modified with FAUX-Ag, while no significant signal was detected with bare electrodes or FAUX-modified electrodes (see Figure 2 and Table S1 in SI), confirming the silver oxidation reaction induced by chloride ions and indicating that only a negligible quantity of chloride ions interacts with the reference electrode (Ag/AgCl). Furthermore, as observed in Figure S2, no silver oxidation peak was observed when the silver cluster–zeolite composite modified electrode was exposed to water, confirming that the probe does not react with chloride ions from the reference electrode.

**Figure 3:**
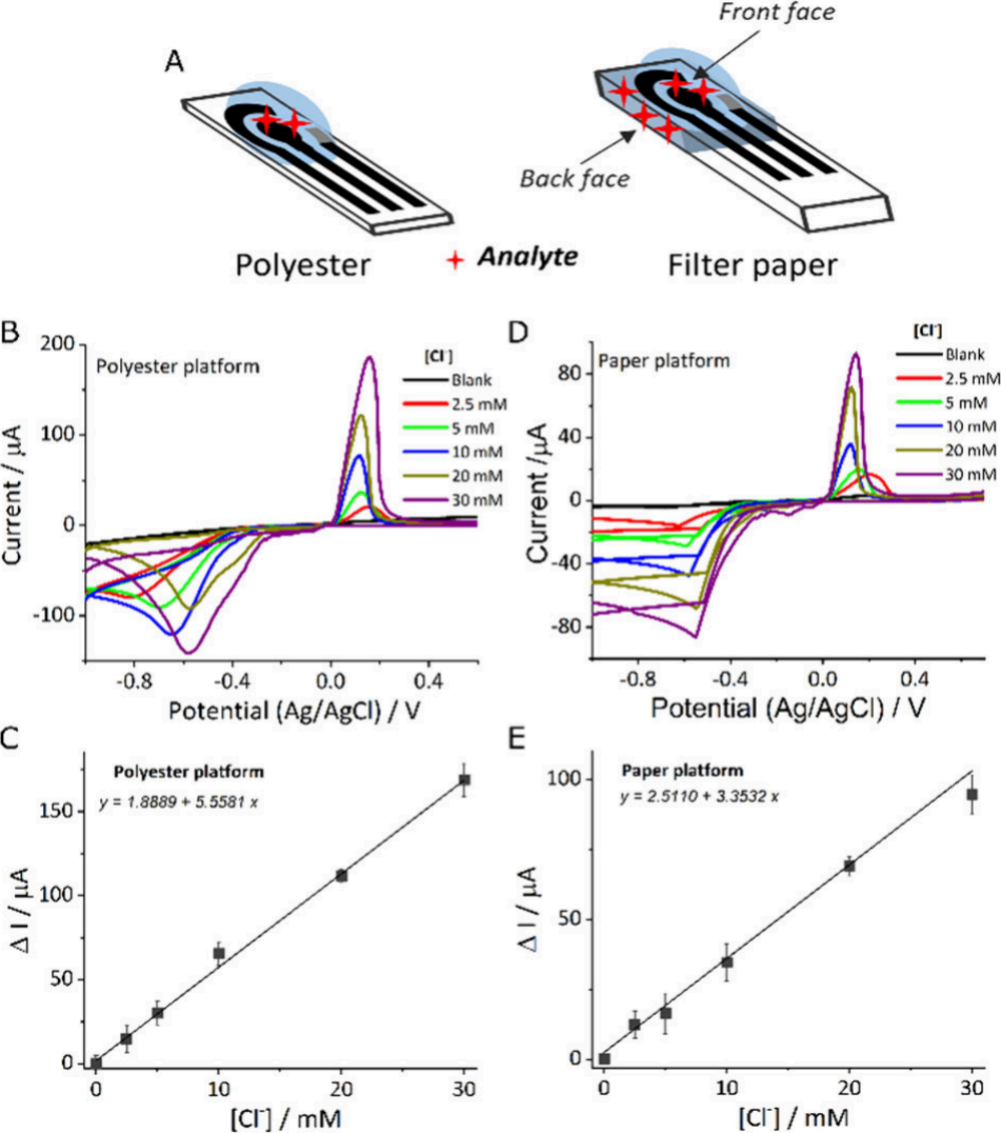
Comparison of polyester and filter paper platforms for sensing with screen-printed electrodes modified with silver clusters embedded within zeolites. (A) Schematic representation of the analyte interaction in polyester and filter paper platforms. (B) Evaluation of the polyester platform through different concentrations of KCl evaluated by a cyclic voltammetry scan rate of 0.05 V/s. (C) Calibration curve of KCl in polyester. (D) Evaluation of filter paper platform through different concentrations of KCl evaluated by a cyclic voltammetry scan rate of 0.05 V/s. (E) Calibration curve of KCl in a filter paper.

To fully characterize the electrochemical system and to provide an alternative in sensing development, polyester- and paper-based electrodes were interrogated in the presence of increasing concentrations of chloride ions using cyclic voltammetry, a standard technique for electrochemical characterization. The electrochemical performance for detecting chloride ions could be influenced by the diverse substrates used for the electrodes. Additionally, to promote more sustainable platforms, particularly paper-based ones capable of storing reagents and filtering matrices, two configurations of electrochemical cells were investigated: one involving the dropping of solution onto a nonporous substrate (polyester) and another utilizing the inherent porosity of paper-based substrates, as illustrated in Figure 3A.

First, the optimal amount of FAUX-Ag decorating the working electrode and the best scan rate were evaluated on polyester-based electrodes by performing a calibration curve with different concentrations of potassium chloride up to 30 mM, quantifying the peak height resulting from silver oxidation (see Supporting Information (SI), Figures S3–S6). The limit of detection, calculated as the interpolation in the calibration curve of the blank plus three times its standard deviation, the coefficient of correlation (*R*^2^), the sensitivity, and the coefficient of variation (CV) were employed to determine the optimal performance. As detailed in Tables S2 and S3 in the SI, the optimal concentration of FAUX-Ag was found to be 6 mg/mL and the best scan rate was 0.05 V/s, yielding a limit of detection of ca. 2.4 mM, *R*^2^ of 0.9962, a slope of 5.56 μA/mM, and a CV of 0.19. The calibration curve constructed under these optimized conditions is displayed in Figure 3B–C.

The surface of the working electrode made on filter paper was modified using the same parameters (using 4 μL of 6 mg/mL FAUX-Ag as the standardized concentration), and the optimal scan rate was also evaluated (see Figures S7–S8 and Table S4 in the SI, as well as Figure 3D–E). Similar to the polyester platform, the optimal scan rate was determined to be 0.05 V/s with a limit of detection of 1.42 mM, an *R*^2^ of 0.9959, a slope of 3.34 μA/mM, and a CV of 0.20.

Comparing the analytical performance of polyester- and paper-based substrates revealed that the paper-based substrate achieved a better detection limit (2.4 mM vs 1.42 mM), possibly due to the high porosity of filter paper and the multilayer deposition, which increased the interaction surface between the electrode and analyte. Conversely, the polyester-based substrate exhibited higher sensitivity (5.56 μA/mM vs 3.34 μA/mM), which may be attributed to the more homogeneous deposition of carbon ink.

Additionally, the stability of the polyester platform-modified electrodes was evaluated by performing a degradation probe over 30 cycles in the presence of 10 mM KCl (see Figure S9, SI). The final response was 84.5% of the initial electrode response, highlighting their reliable stability, which is particularly advantageous for disposable devices.

### 3.2 Electrocatalytic Character of the Composite: H_2_O_2_ and Glucose Sensing

Besides their oxidative–reductive potential, silver clusters have demonstrated high electrocatalytic activity in certain electrochemical reactions, such as H_2_O_2_ reduction.^16,29^ However, the combination of silver clusters in zeolites with disposable electrodes has not yet been characterized.

**Figure 4:**
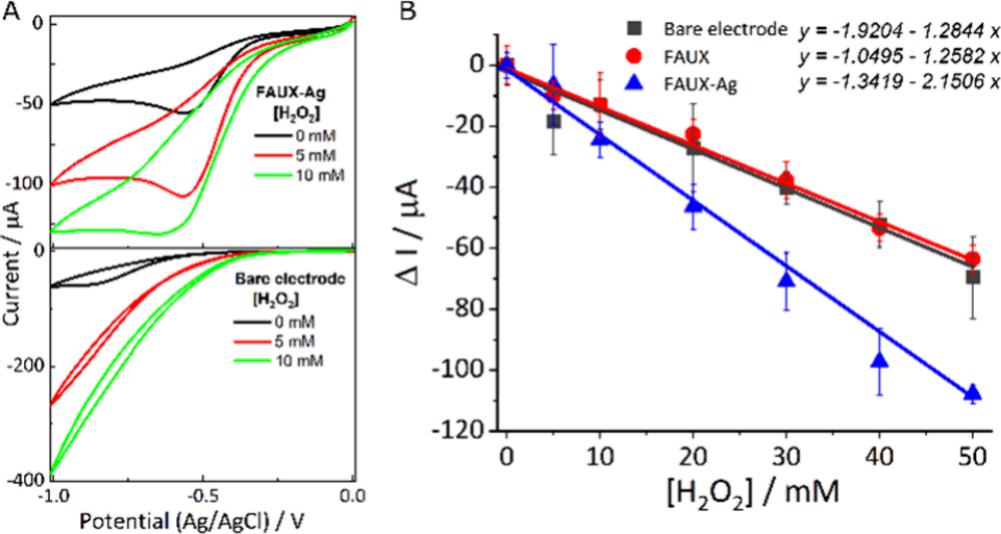
Comparison of the bare working electrode and the modified working electrode. (A) Cyclic voltammetry. (B) Calibration curve of bare electrode, electrode modified with FAUX, and electrode modified with FAUX-Ag measured by chronoamperometry at 60 s. Applied potential: −0.7 V.

To evaluate the H_2_O_2_ reduction reaction, cyclic voltammetry was performed, comparing bare electrodes with those decorated with zeolites embedded in silver clusters (FAUX-Ag). As observed in Figure 4A, a peak around −0.6 V relative to H_2_O_2_ reduction was observed with the FAUX-Ag modified electrode,^30^ with a decrease in current corresponding to increasing concentrations of H_2_O_2_.

Contrary to the modified electrode, bare electrodes did not produce any detectable peaks. To assess the ability to reduce H_2_O_2_, chronoamperometry was conducted using both modified and unmodified electrodes at various H_2_O_2_ concentrations. As displayed in Figure 4B and detailed in Table S5 of the SI, the presence of zeolite improved the limit of detection and reduced the CV, as its cavities can host the analyte, enhancing the interaction between the analyte and the electrode. However, the best sensitivity and limit of detection were achieved with the FAUX-Ag modified electrode, attributable to its electrocatalytic effect.

Different amounts of FAUX-Ag and different applied potentials for chronoamperometry were tested as illustrated in Figures S10–S11 and Tables S6–S7 in the SI. The optimal concentration was determined to be 6 mg/mL, with −0.7 V as the most favorable potential, resulting in a limit of detection of ca. 5.3 mM, a slope of −2.1 mM/μA, a *R*^2^ of 0.9961, and a CV of −0.45.

Once the capability of FAUX-Ag clusters to selectively detect H_2_O_2_ via electrocatalysis was demonstrated, the same system was further explored for developing a glucose biosensor using glucose oxidase enzyme, which generates H_2_O_2_ as a byproduct of glucose oxidation.^31^

**Figure 5:**
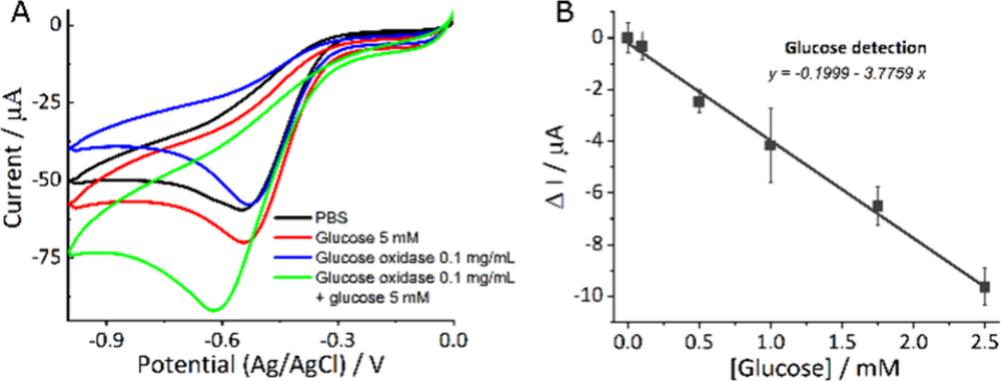
(A) Response of PBS, glucose, glucose oxidase, and glucose oxidase with glucose. (B) Calibration curve of glucose detection.

To corroborate that the peak obtained is related to the enzymatic reaction, the responses of PBS, glucose, glucose oxidase, and a mixture of glucose oxidase with glucose after 5 min of reaction (all these solutions prepared in PBS) were evaluated by cyclic voltammetry. As observed in Figure 5A, in the presence of PBS, glucose, and glucose oxidase solutions, a reduced peak appeared centered around 0.5 V, associated with O_2_ reduction,^32^ whereas in the enzymatic reaction, a more intense peak at −0.6 V was attributed to H_2_O_2_ reduction.

Chronoamperometry was then used to construct a calibration curve to determine the optimal concentration of glucose oxidase, testing three concentrations (0.1, 1, and 10 mg/mL). As shown in Figure S13 in the SI, a saturation point was reached after 2.5 mM with both 1 and 10 mg/mL glucose oxidase, while 0.1 mg/mL did not produce a significant signal. Based on the parameters of SI Table S8, the optimal glucose oxidase concentration was determined to be 10 mg/mL. A calibration curve in the linear range (0 to 2.5 mM) was constructed, evaluating the applied potential for chronoamperometry; see Figure 5B and SI Figure S14 and Table S9. Obtaining −0.6 V as the best-applied potential resulted in a limit of detection of 0.4 mM, a slope of −3.78 μA/mM, an *R*^2^ of 0.9902, and a CV of −0.48.

The selectivity of the biosensing platform was evaluated in the presence of common interfering substances found in human fluids, including cholesterol, ascorbic acid, and uric acid. All the interfering species were tested at a concentration of 2 mM. As displayed in Figure S15 in the SI, the current intensity for cholesterol and ascorbic acid was comparable to the blank. Nevertheless, the current signal for uric acid at 2 mM decreased noticeably, while at 1 mM, it was similar to the blank. This concentration exceeds physiological levels in human fluids,^33^ confirming the potential for designing a novel platform for healthcare applications. This platform promotes a simple and low-cost modification in comparison to other methodologies reported that require more complex surface modifications of the working electrode,^34,35^ proposing for future works the exploration of the immobilization of enzymes or other target molecules within the zeolite pores.

## 4 Conclusion

Zeolites embedding silver clusters were demonstrated as a novel and active material for modifying portable and printed strips. This composite enhances analyte immobilization on the electrode surface, increasing the electrical conductivity and improving the electron exchange transfer in the electroactive surface area. A sensitivity enhancement of 64% was achieved compared to the bare electrode, resulting in a portable electrochemical sensor with high accuracy and selectivity in the presence of clinically relevant molecules, particularly those present in human fluids such as cholesterol, ascorbic acid, and uric acid. The versatile role of the silver cluster–zeolite composite, without further modifications, was evident in its ability to detect analytes of different natures using two mechanisms, its oxidative–reductive potential for ion detection and its electrocatalyst activity for (bio)molecule reduction, positioning silver cluster–zeolite composites as promising materials for surface modification of electrochemical strips toward the detection of clinically and environmentally relevant molecules. Thus, this contributes to the development of electroactive materials used in miniaturized electrochemical sensors for portable platforms and point-of-care devices.

## Supporting Information Available

Morphological studies of modified and nonmodified electrodes, cyclic voltammetry experiments, calibration curves of both chloride ions and glucose, and optimization parameters of both cyclic voltammetry and amperometry measurements (PDF)
