# Supplementary material for: Electrochemical Strips Modified with Zeolites Embedding Silver Clusters for Versatile (Bio)Systems
Source: Anal Chem. 2024 Oct 29;96(45):17915–21. doi: 10.1021/acs.analchem.4c03654 (PMC11561877; doi:10.1021/acs.analchem.4c03654)
Supplement: Supplementary file 1 — ac4c03654_si_001.pdf [file ac4c03654_si_001.pdf]

Supporting information

Electrochemical Strips Modified with Zeolites Embedding Silver Clusters for Versatile (Bio)Systems

Cecilia García-Guzmán[a,b], Ada Raucci[a], Eduardo Coutino-Gonzalez[c]*, Eden Morales-Narváez[d]*, and Stefano Cinti[a,e,f]*

[a] Department of Pharmacy, University of Naples "Federico II", 80131 Naples, Italy
[b] Centro de Investigaciones en Óptica, A. C., Loma del Bosque 115, Lomas del Campestre, León, Guanajuato, 37150 Mexico.
[c] Materials & Chemistry Unit (MatCh), VITO, Flemish Institute for Technological Research, Boeretang 200, Mol, B-2400 Belgium.
[d] Biophotonic Nanosensors Laboratory, Centro de Física Aplicada y Tecnología Avanzada (CFATA), Universidad Nacional Autónoma de México (UNAM), Querétaro, 76230 Mexico.
[e] Bioelectronics Task Force at University of Naples Federico II, Via Cinthia 21, Naples 80126, Italy.
[f] Sbarro Institute for Cancer Research and Molecular Medicine, Center for Biotechnology, College of Science and Technology, Temple University, Philadelphia, PA 19122, USA

Table of contents

Figures

Figure S1. EDS evaluation of the electrode surface on the polyester substrate after modification with silver clusters embedded within zeolites.

Figure S2. Evaluation of polyester platform through water and different concentrations of KCl evaluated by cyclic voltammetry scan rate 0.05 V/s, [FAUX-Ag] 6 mg/mL.

Figure S3. Evaluation of different concentrations of FAUX-Ag to modify the working electrode surface. Cyclic voltammetry of silver oxidation by chloride ions detection. Polyester platform. Scan rate 0.05 V.

Figure S4. Evaluation of different concentrations of FAUX-Ag to modify the working electrode surface. Calibration curve of silver oxidation by chloride ions detection. Polyester platform. Scan rate 0.05 V.

Figure S5. Evaluation of different scan rates applied. Cyclic voltammetry of silver oxidation by chloride ions detection. Polyester platform. [FAUX-Ag] 6 mg/mL.

Figure S6. Evaluation of different scan rates. Calibration curve of silver oxidation by chloride ions detection. Polyester platform. [FAUX-Ag] 6 mg/mL.

Figure S7. Evaluation of different scan rates applied. Cyclic voltammetry of silver oxidation by chloride ions detection. Paper platform. [FAUX-Ag] 6 mg/mL.

Figure S8. Evaluation of different scan rates. Calibration curve of silver oxidation by chloride ions detection. Paper platform. [FAUX-Ag] 6 mg/mL.

Figure S9. Cyclic voltammograms of initial conditions and after 30 cycles of degradation probe (Scan rate 100 mV/s). Polyester platform, [FAUX-Ag] 6 mg/mL, [KCl] 10 mM, scan rate 50 mV/s.

Figure S10. Evaluation of different concentrations of FAUX-Ag to modify the working electrode for hydrogen peroxide (H_2O_2) detection. Calibration curve constructed measured by chronoamperometry at 60 seconds. Applied potential -0.6 V.

Figure S11. Evaluation of different applied potentials for hydrogen peroxide (H_2O_2) detection. Calibration curve obtained by chronoamperometry at 60 seconds. [FAUX-Ag] 6 mg/mL.

Figure S12. Evaluation of different concentrations of glucose oxidase. Cyclic voltammetry for the determination of hydrogen peroxide oxidation peak after 5 minutes of the enzymatic reaction. [FAUX-Ag] 6 mg/mL, scan rate 0.05 V.

Figure S13. Evaluation of different glucose oxidase concentrations for glucose detection by the enzymatic reaction. Calibration curve obtained by chronoamperometry at 60 seconds after 5 minutes of the enzymatic reaction. [FAUX-Ag] 6 mg/mL, applied potential -0.6 V.

Figure S14. Evaluation of different applied potentials for glucose detection. Calibration curve obtained by chronoamperometry at 60 seconds after 5 minutes of the enzymatic reaction. [FAUX-Ag] 6 mg/mL, [glucose oxidase] 10 mg/mL.

Figure S15. Interference evaluation for glucose detection. Measures taken at 60 seconds of chronoamperometry after 5 minutes of the enzymatic reaction. [FAUX-Ag] 6 mg/mL, [Glucose oxidase] 10 mg/mL, applied potential -0.6 V.

Tables

Table S1. Intensity of the oxidation peak by using different electrode surface modifications.

Table S2. Evaluation of different [FAUX-Ag] for modifying the working electrode. Polyester platform.

Table S3. Evaluation of different scan rates. Polyester platform. Working electrode modified with [FAUX-Ag] 6 mg/mL.

Table S4. Evaluation of different scan rates. Paper platform. Working electrode modified with [FAUX-Ag] 6 mg/mL.

Table S5. Comparison of bare electrode, FAUX, FAUX-Ag for hydrogen peroxide detection.

Table S6. Evaluation of different FAUX-Ag concentrations for hydrogen peroxide detection.

Table S7. Evaluation of different applied potentials for hydrogen peroxide detection. [FAUX-Ag] 6 mg/mL.

Table S8. Evaluation of different concentrations of glucose oxidase for glucose detection after 5 minutes of reaction. Calibration curve made from 0 to 10 mM (complete curve).

Table S9. Evaluation of different applied potentials for glucose detection after 5 minutes of enzymatic reaction. Calibration curve from 0 to 2.5 mM (linear range). [FAUX-Ag] 6 mg/mL, [Glucose oxidase] 10 mg/mL.

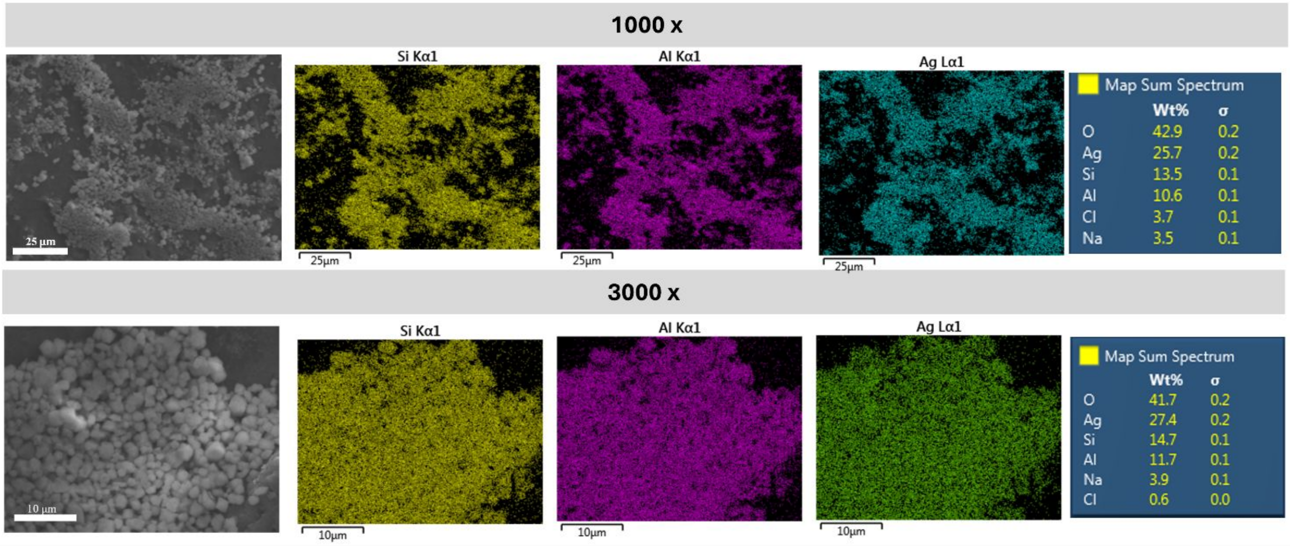

Figure S1. EDS evaluation of the electrode surface on the polyester substrate after modification with silver clusters embed within zeolites.

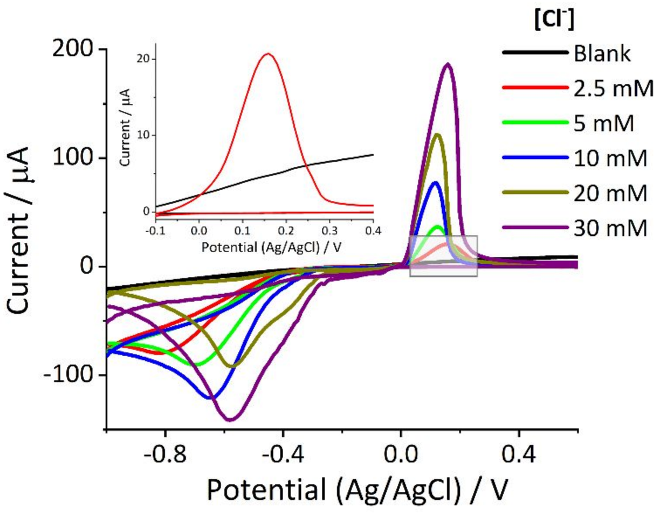

Figure S2. Evaluation of polyester platform through water and different concentrations of KCl evaluated by cyclic voltammetry scan rate 0.05 V/s, [FAUX-Ag] 6 mg/mL.

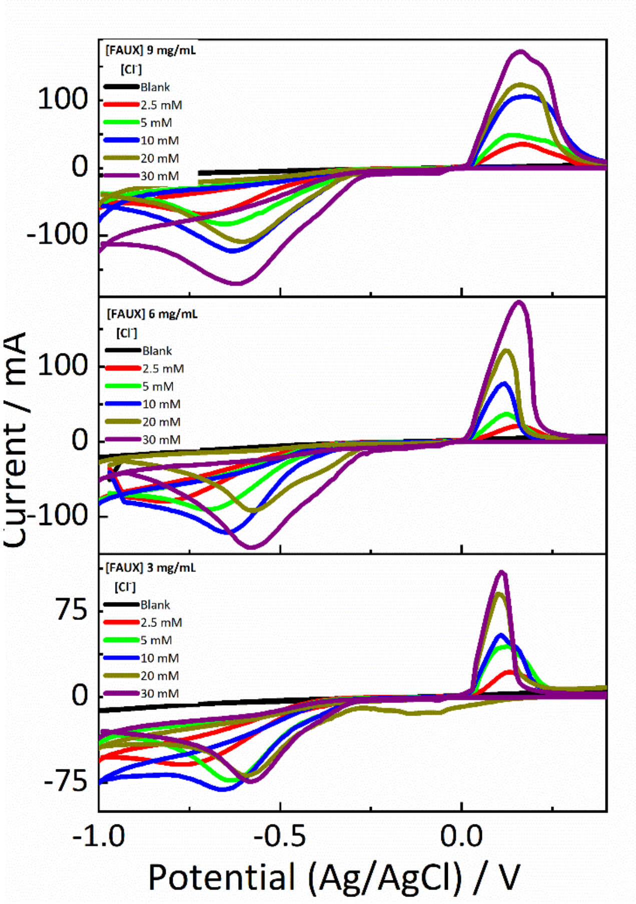

Figure S3. Evaluation of different concentrations of FAUX-Ag to modify the working electrode surface. Cyclic voltammetry of silver oxidation by chloride ions detection. Polyester platform. Scan rate 0.05 V.

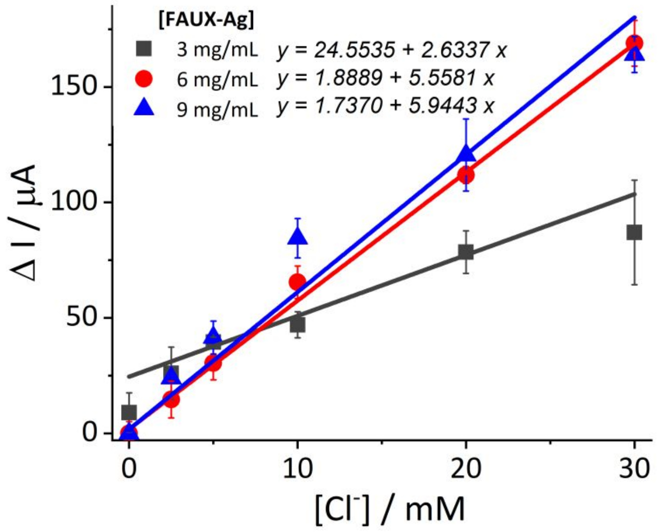

Figure S4. Evaluation of different concentrations of FAUX-Ag to modify the working electrode surface. Calibration curve of silver oxidation by chloride ions detection. Polyester platform. Scan rate 0.05 V.

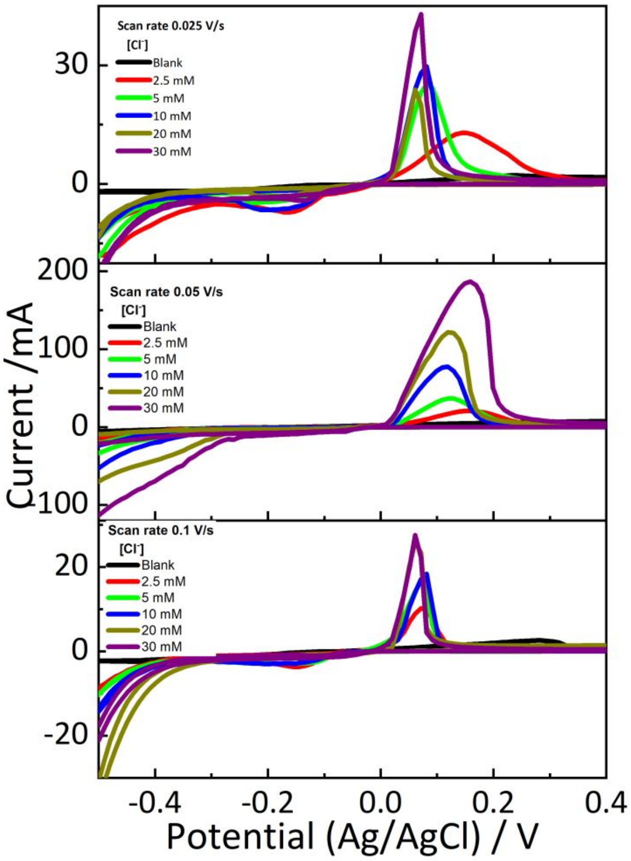

Figure S5. Evaluation of different scan rates applied. Cyclic voltammetry of silver oxidation by chloride ions detection. Polyester platform. [FAUX-Ag] 6 mg/mL.

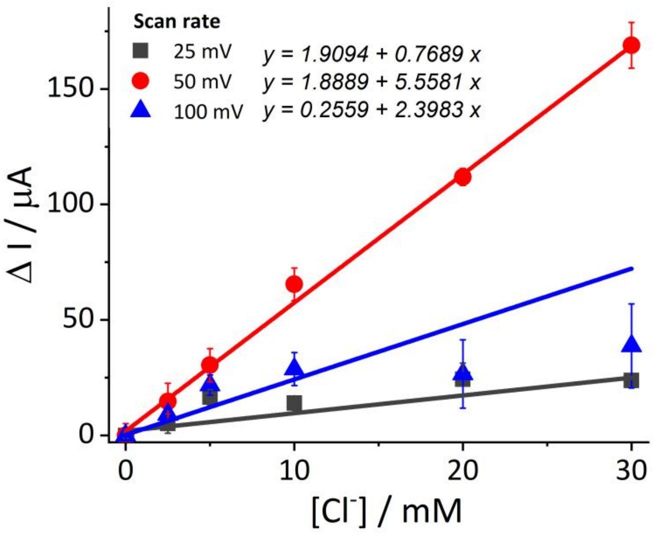

Figure S6. Evaluation of different scan rates. Calibration curve of silver oxidation by chloride ions detection. Polyester platform. [FAUX-Ag] 6 mg/mL.

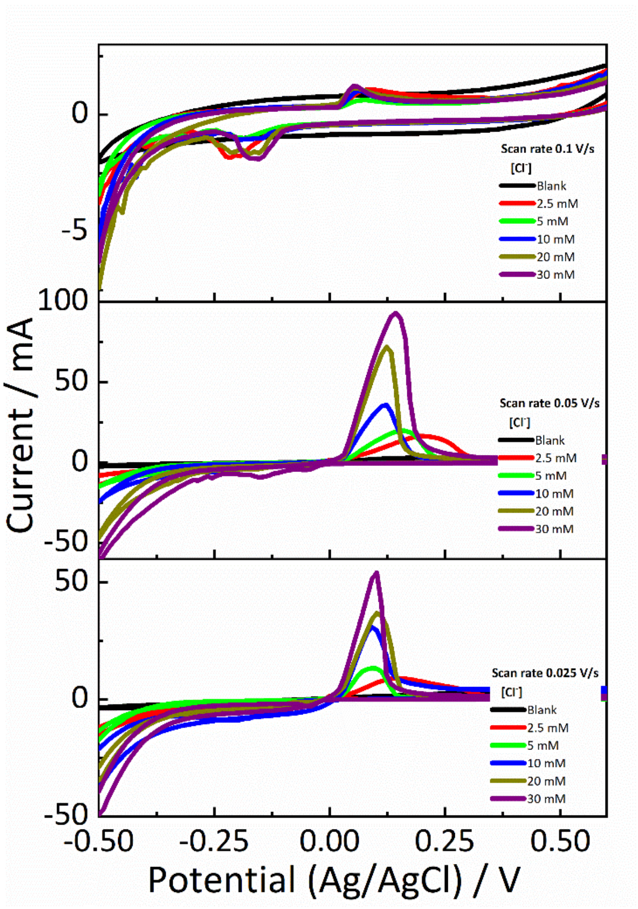

Figure S7. Evaluation of different scan rates applied. Cyclic voltammetry of silver oxidation by chloride ions detection. Paper platform. [FAUX-Ag] 6 mg/mL.

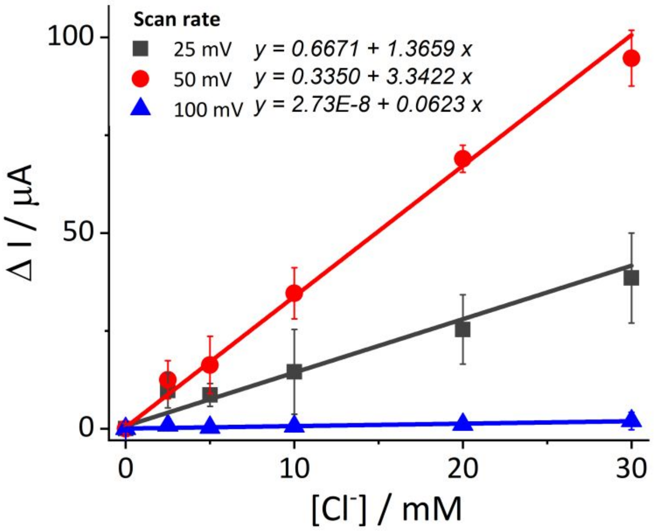

Figure S8. Evaluation of different scan rates. Calibration curve of silver oxidation by chloride ions detection. Paper platform. [FAUX-Ag] 6 mg/mL.

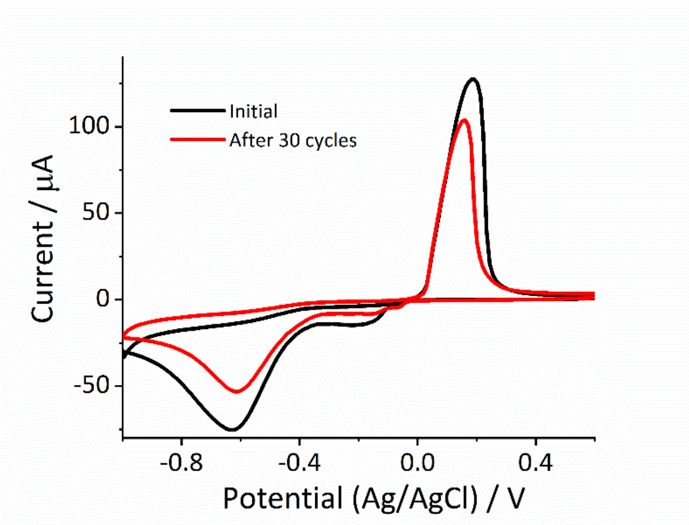

Figure S9. Cyclic voltammograms of initial conditions and after 30 cycles of degradation probe (Scan rate 100 mV/s). Polyester platform, [FAUX-Ag] 6 mg/mL, [KCl] 10 mM, scan rate 50 mV/s.

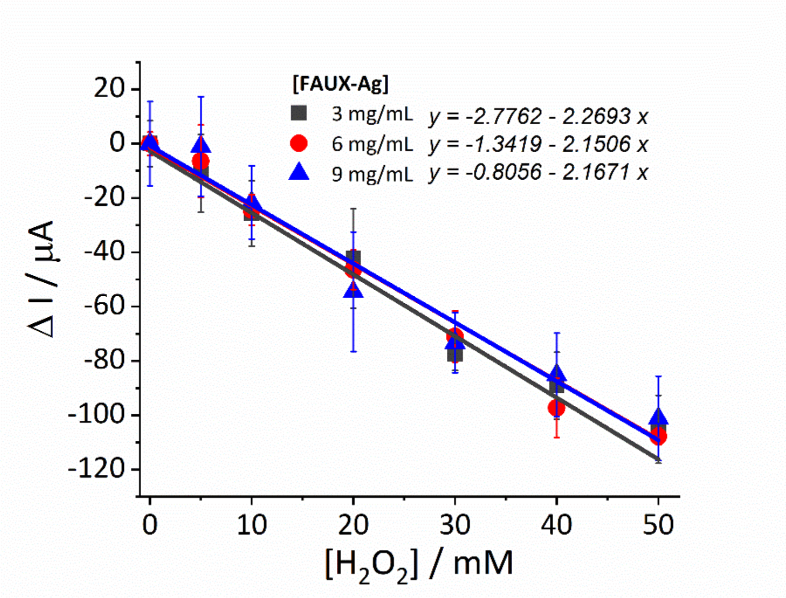

Figure S10. Evaluation of different concentrations of FAUX-Ag to modify the working electrode for hydrogen peroxide (H_2O_2) detection. Calibration curve constructed measured by chronoamperometry at 60 seconds. Applied potential -0.6 V.

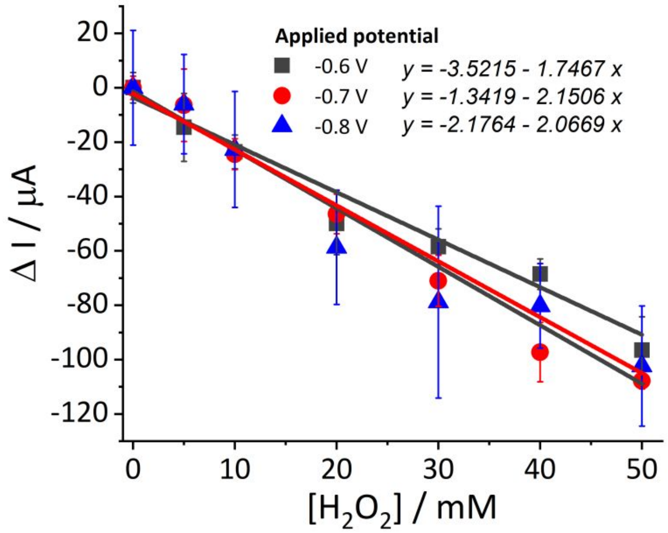

Figure S11. Evaluation of different applied potentials for hydrogen peroxide (H_2O_2) detection. Calibration curve obtained by chronoamperometry at 60 seconds. [FAUX-Ag] 6 mg/mL.

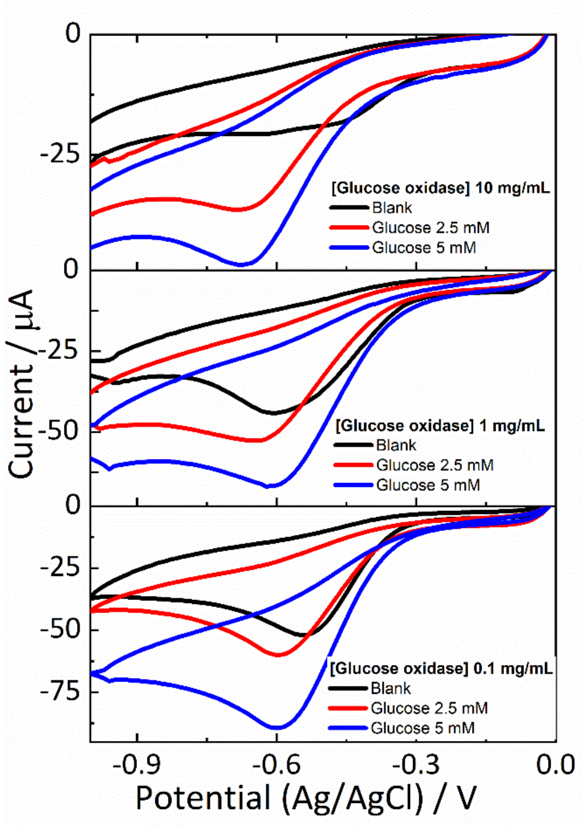

Figure S12. Evaluation of different concentrations of glucose oxidase. Cyclic voltammetry for the determination of hydrogen peroxide oxidation peak after 5 minutes of the enzymatic reaction. [FAUX-Ag] 6 mg/mL, scan rate 0.05 V.

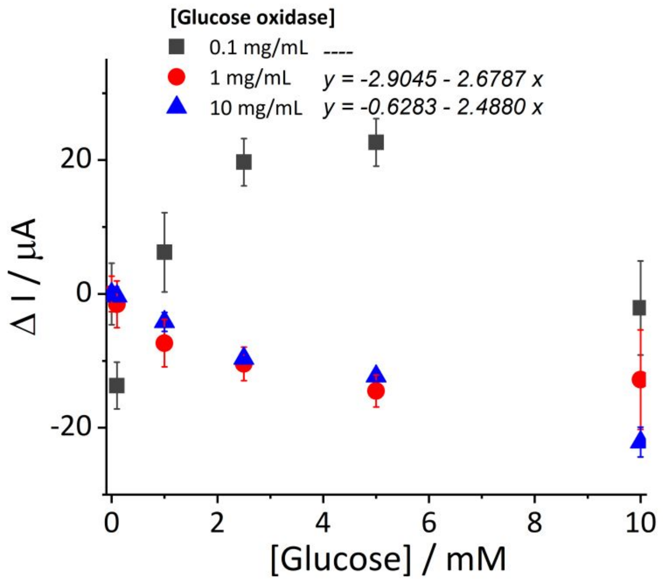

Figure S13. Evaluation of different glucose oxidase concentrations for glucose detection by the enzymatic reaction. Calibration curve obtained by chronoamperometry at 60 seconds after 5 minutes of the enzymatic reaction. [FAUX-Ag] 6 mg/mL, applied potential -0.6 V.

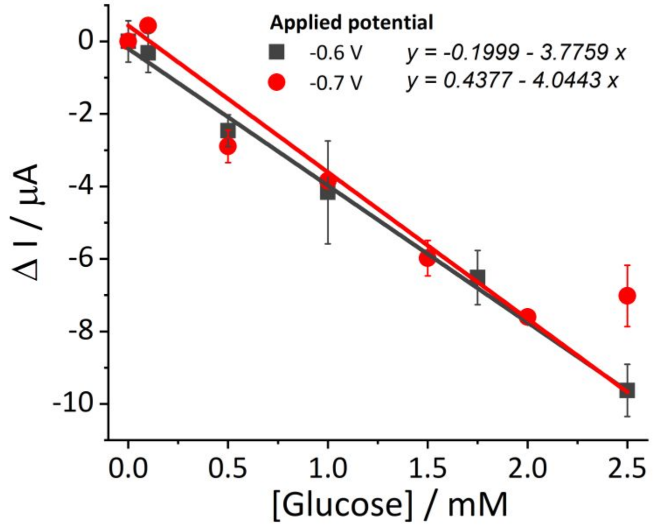

Figure S14. Evaluation of different applied potentials for glucose detection. Calibration curve obtained by chronoamperometry at 60 seconds after 5 minutes of the enzymatic reaction. [FAUX-Ag] 6 mg/mL, [glucose oxidase] 10 mg/mL.

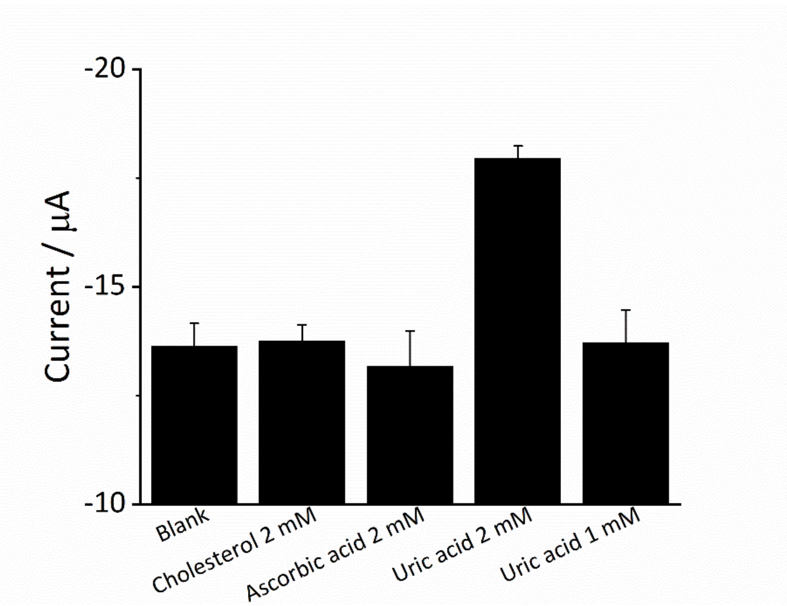

Figure S15. Interference evaluation for glucose detection. Measures taken at 60 seconds of chronoamperometry after 5 minutes of the enzymatic reaction. [FAUX-Ag] 6 mg/mL, [Glucose oxidase] 10 mg/mL, applied potential -0.6 V.

Tables

Table S1. Intensity of the oxidation peak by using different electrode surface modifications.

Electrode modification	***Current (µA)***
Bare electrode	0.41
FAUX	0.47
FAUX-Ag	77.49

Table S2. Evaluation of different [FAUX-Ag] for modifying the working electrode. Polyester platform.

[FAUX-Ag] (mg/mL)	***Limit of detection (mM)***	***Slope (µA/mM)***	***R^2***	***CV***
3	4.34623	2.63366	0.82763	0.33257
6	**2.39493**	**5.55808**	**0.99618**	**0.19511**
9	0.21247	5.94432	0.94826	0.11111

Table S3. Evaluation of different scan rates. Polyester platform. Working electrode modified with [FAUX-Ag] 6 mg/mL.

Scan rate (mV/s)	***Limit of detection (mM)***	***Slope (µA/mM)***	***R^2***	***CV***
25	5.31469	0.76885	0.85956	0.31625
50	**2.39493**	**5.55808**	**0.99618**	**0.19511**
100	1.56920	2.39834	0.75094	0.31941

Table S4. Evaluation of different scan rates. Paper platform. Working electrode modified with [FAUX-Ag] 6 mg/mL.

Scan rate (mV/s)	*Limit of detection (mM)*	*Slope (µA/mM)*	*R^2*	*CV*
25	3.73580	1.36594	0.90170	0.43791
50	**1.42283**	**3.34220**	**0.99588**	**0.20069**
100	0.00332	0.06228	0.69282	0.53809

Table S5. Comparison of bare electrode, FAUX, FAUX-Ag for hydrogen peroxide detection.

Functionalization	*Limit of detection (mM)*	*Slope (µA/mM)*	*R^2*	*CV*
Bare electrode	13.68692	-1.28441	0.97221	-0.36689
FAUX	14.11614	-1.25817	0.98905	-0.29970
FAUX-Ag	**5.33138**	**-2.15061**	**0.99607**	**-0.45370**

Table S6. Evaluation of different FAUX-Ag concentrations for hydrogen peroxide detection.

[FAUX-Ag] (mg/mL)	*Limit of detection (mM)*	*Slope (µA/mM)*	*R^2*	*CV*
3	9.88061	-2.26934	0.97134	-0.42466
6	**5.33139**	**-2.15061**	**0.99607**	**-0.45370**
9	21.02273	-2.16708	0.96287	-3.21995

Table S7. Evaluation of different applied potentials for hydrogen peroxide detection. [FAUX-Ag] 6 mg/mL.

Applied potential (V)	*Limit of detection (mM)*	*Slope (µA/mM)*	*R^2*	*CV*
-0.6	7.58131	-1.74668	0.97224	-0.30883
-0.7	**5.33139**	**-2.15061**	**0.99607**	**-0.45370**
-0.8	29.74205	-2.05585	0.95134	-0.86293

Table S8. Evaluation of different concentrations of glucose oxidase for glucose detection after 5 minutes of reaction. Calibration curve made from 0 to 10 mM (complete curve).

Glucose oxidase concentration (mg/mL)	*Limit of detection (mM)*	*Slope (µA/mM)*	*R^2*	*CV*
0.1	---	---	---	-0.46198
1	2.41759	-2.10352	0.69820	-0.74360
10	**0.43852**	**-2.48800**	**0.92377**	**-0.45776**

Table S9. Evaluation of different applied potentials for glucose detection after 5 minutes of enzymatic reaction. Calibration curve from 0 to 2.5 mM (linear range). [FAUX-Ag] 6 mg/mL, [Glucose oxidase] 10 mg/mL.

Applied potential (V)	*Limit of detection (mM)*	*Slope (µA/mM)*	*R^2*	*CV*
- 0.6 V	**0.40243**	**-3.77589**	**0.99023**	**-0.48688**
- 0.7 V	0.19867	-4.04433	0.97178	-0.25820
